# The Modification of Poly(3-Hydroxybutyrate-co-4-hydroxybutyrate) by Melt Blending

**DOI:** 10.3390/polym14091725

**Published:** 2022-04-23

**Authors:** Minki Jo, Yunjae Jang, Eunhye Lee, Sooan Shin, Ho-Jong Kang

**Affiliations:** 1 Department of Polymer Science and Engineering, Dankook University, 152 Jukjeon-ro, Suji-gu, Yongin-si 16890, Gyeonggi-do, Korea; mc0281@naver.com; 2 CJ Cheiljedang Corporation, 55, Gwanggyo-ro 42beon-gil, Yeongtong-gu, Suwon-si 16495, Gyeonggi-do, Korea; yunjae.jang@cj.net (Y.J.); eunhye.lee@cj.net (E.L.); sooanshin@gmail.com (S.S.)

**Keywords:** P(3HB-co-4HB), 4HB contents, mixtures, thermal properties, rheological properties, mechanical properties

## Abstract

Crystalline and noncrystalline poly(3-hyroxybutylate-co-4-hyroxybutylate) (P(3HB-co-4HB)) were melt blended to obtain mixtures of P(3HB-co-4HB) copolymers. The mixtures and P(3HB-co-4HB) copolymers of different 4HB contents were compared to study the effect of 4HB content on the properties of the copolymers and mixtures. P(3HB-co-4HB) copolymer mixtures, having various 4HB content, have been successfully made by melt blending instead of bacterial biosynthesis. In the case of copolymers, they were noncrystalline when the 4HB content was over 16%, while the P(3HB-co-4HB) mixtures at the same 4HB content were crystalline. The mixtures had a higher glass transition temperature, suggesting that their chain mobility is relatively low compared with the copolymer having the same 4HB content. Due to this effect, the mixture is expected to have a higher melt viscosity and a lower loss tangent to exhibit better melt processing properties. The mechanical properties of the mixtures show a similar behavior to the copolymers in that the tensile strength and the modulus decreases and elongation at the break increases with an increase in the 4HB content.

## 1. Introduction

Polyhydroxyalkanoate (PHA) is a biodegradable polyester produced by microorganisms and has the benefit of being degradable by microorganisms under a diverse environment [1,2,3,4,5,6] contrary to the chemically manufactured polylactic acid (PLA) [7,8] and polybutylene succinate (PBS) [9,10]. Poly 3-hydroxybutyrate(P3HB), a typical PHA, is highly crystalline and its crystallization rate is very slow due to its stereoregular chemical structure. Therefore, the spherulites formed are very large and spherulite/spherulite interfaces are formed where fracture occurs, making it brittle [11,12,13,14]. Degradation occurs easily above the processing temperature of 180 °C, giving it the drawback of a very narrow melt processing window [15,16].

To resolve the drawback, control of the crystallization rate and degradation properties by melt blending with diverse biodegradable polymers such as PLA and PBS, etc., is being carried out [17,18,19]. Utilization of the chemical structure of P(3HB) to induce reactions such as carboxylation, halogenation, hydroxylation, grafting, epoxidation, etc., by adding diverse chemicals to control the properties through structural changes such as branching and crosslinking, etc., is possible [20,21,22,23,24]. Along with this method, the most widely used method to control the properties of P(3HB) is bacterial synthesis of diverse copolymers. Representative copolymers are P(3HB-co-3HV) [25,26], P(3HB-co-HDD) [27], etc., and recently research has focused on P(3HB-co-4HB) [28,29,30,31,32].

When 4HB is added to the biosynthesis of P(3HB), a P(3HB-co-4HB) copolymer is formed and the flexibility of the 4HB segment decreases the brittle character of P(3HB) along with decrease in the glass transition temperature, decrease in the melting temperature, and decrease in the crystallization rate and crystallinity. When the 4HB content exceeds 20%, the crystallization of P(3HB-co-4HB) is difficult [33], and as a result, modulus and tensile strength decreases and elongation at break increases. Furthermore, the melt processibility decreases significantly due to the decrease in melt viscosity and increase in the loss tangent. Besides the effect of the 4HB segment on the physical properties of the P(3HB-co-4HB) copolymer, the 4HB segment causes an enhancement of thermal stability of P(3HB). The thermal degradation of P(3HB-co-4HB) occurs due to the random unzipping degradation of 3HB, similar to P(3HB), however, 4HB is hardly activated for degradation due to the methylene group to stop unzipping degradation [34]. The cyclization and transesterification of degraded 4HB also results in the improvement of thermal stability of P(3HB-co-4HB).

To resolve the problem of crystallinity change and the decrease in melt processibility with an increase in 4HB content in P(3HB-co-4HB), crystalline P(3HB-co-4HB) and noncrystalline P(3HB-co-4HB) is melt blended to prepare mixtures. The change in the crystallization behavior and the melt processibility with an increase in 4HB content in the mixture is compared with P(3HB-co-4HB) of similar 4HB content.

## 2. Experimental

### 2.1. Materials and Mixture Preparation

The copolymer of 3-hyroxybutyrate and 4-hyroxybutyrate, poly(3-hyroxybutylate-co-4-hyroxybutylate), was provided by CJ Cheiljedang and their 4HB content and molecular weights are shown in Table 1. To enhance the properties of P(3HB-co-4HB) copolymer, crystalline P(3HB-co-10% 4HB) and noncrystalline P(3HB-co-53.7% 4HB) were melt blended at composition ratios of 9/1–4/6 in an internal mixer (Haake, Rheomix600, Vreden, Germany). To minimize degradation during the melt blending process, they were blended at 140 °C and 20 rpm for 10 min. All P(3HB-co-4HB) samples were processed in the same way to give the same thermal history. The P(3HB-co-4HB) and P(3HB-co-4HB) mixtures thus obtained were put in 1T thick 150 mm × 150 mm mold in a compression molding machine (QMESYS, QM900A, Anyang, Korea) and kept at 140 °C for 2 min, pressure raised to 8 MPa, then quenched in a water tank at 25 °C for 2 min, to prepare the 1T thick films.

### 2.2. The 4HB Contents of P(3HB-co-4HB) Mixtures

^1^H NMR (Jeol, Jeol400, Tokyo, Japan) was used to determine the 4HB content of the P(3HB-co-10% 4HB) and P(3HB-co-53.7% 4HB) mixtures. The NMR samples were prepared by dissolving CDCl_3_. The 4.1 ppm peak due to the two hydrogens on the number 8 carbon (4HB) and the 1.25 ppm peak due to the three hydrogens on the number 4 carbon (3HB) were compared to calculate the relative presence of the 3HB and 4HB units and calculate the 4HB content.

### 2.3. Thermal Properties of P(3HB-co-4HB) Mixtures

Differential scanning calorimeter (TA, Q20, New Castle, DE, USA) was used to study the effect of 4HB content on the thermal properties of the P(3HB-co-4HB) and P(3HB-co-4HB) mixtures. The effect of 4HB content on the changes in the crystallization temperature and enthalpy in the cooling stage and the glass transition temperature, melting temperature, and the enthalpy of melting in the second heating stage were evaluated by scanning in the range −50~250 °C at the heating and cooling rates of 20 °C/min.

### 2.4. Rheological Properties of P(3HB-co-4HB) Mixtures

The rheological properties of P(3HB-co-4HB) and their mixtures were evaluated on a rotational rheometer (TA, AR200EX, New Castle, DE, USA) with 25 mm diameter samples in the oscillation mode at angular frequencies of 0.1~600 rad/s and the % strain set at 0.1, 3, and 5 at 140 °C, 160 °C, and 180 °C, respectively, to obtain the changes in the complex viscosity (η*) and loss tangent (tanδ) with frequency change. Using obtained zero shear viscosity (η_0_), the curves of ln(η_0_) versus 1/T were plotted and the dependence of P(3HB-co-4HB) and their mixtures shear viscosity on temperature can be described by the Arrhenius equation.

ln(η_0_) = A exp (Ea/RT) (1)

where A is constant related to melt viscosity, and Ea is the activation energy for viscous flow.

### 2.5. Mechanical Properties of P(3HB-co-4HB) Mixtures

The tensile strength, Young’s modulus and elongation at break were measured on a tensile tester (LLOYD, LR-30K, West Sussex, UK) with 1T thick 10 mm × 50 mm samples gripping the top and bottom 10 mm on the jig and extending at 50 mm/min until break.

## 3. Results and Discussion

### 3.1. 4HB Contents of P(3HB-co-4HB) Mixtures

**Figure 1:**
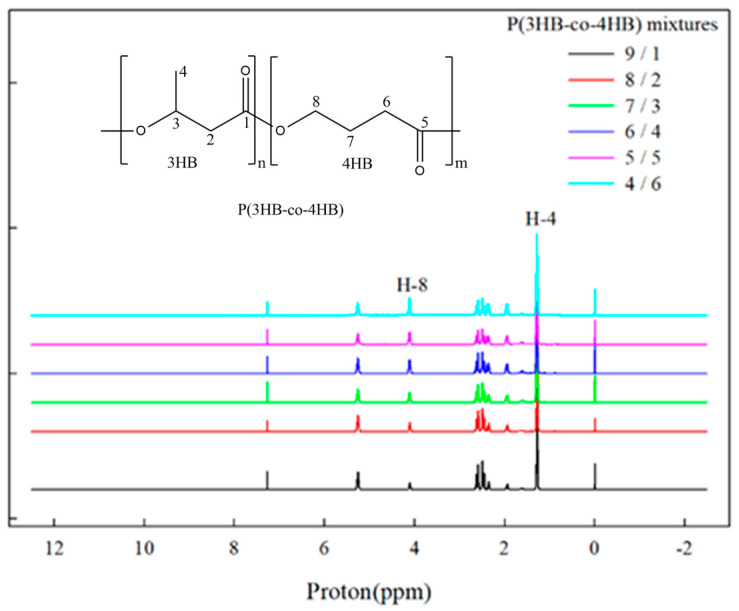
^1^H NMR spectra of P(3HB-co-4HB) mixtures.

**Figure 2:**
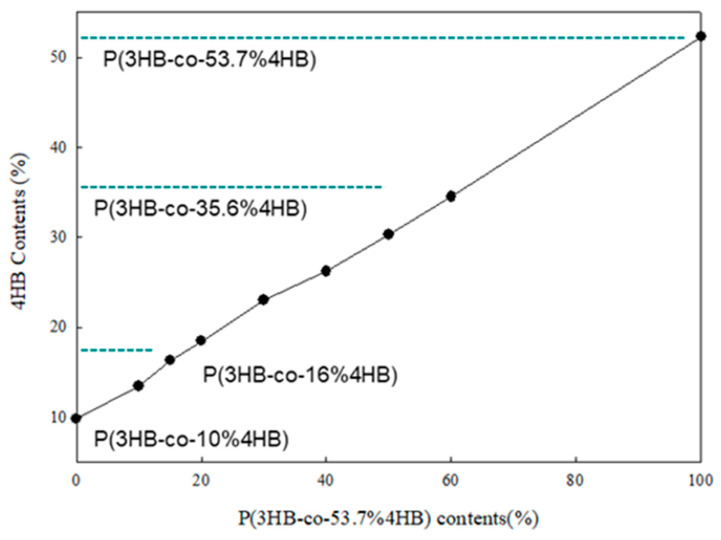
The 4HB contents of P(3HB-co-4HB) mixtures calculated from NMR data and the theoretical line calculated from the composition.

Figure 1 shows the ^1^H NMR spectra of the P(3HB-co-4HB) mixtures of different blend ratios. The peak areas of the 4.1 ppm and 1.25 ppm due to 4HB and 3HB units, respectively, change with the composition of the mixture. The 4HB contents calculated from the ^1^H NMR peak areas are shown in Figure 2 along with the line for the theoretical 4HB content calculated from the blend ratios. The 4HB contents calculated from the NMR data conform to the theoretical values calculated from the blend ratios and P(3HB-co-4HB) mixtures, having 14.4–45% 4HB content that could be prepared. Conformance of the data and the theoretical values suggest that there is no thermal degradation or chemical change in the melt blending process changing the 4HB or 3HB contents. However, only our ^1^H NMR results did not convince us that there was no chemical change in the melt blending, and we may need further study to find out the light cross-linking in the copolymer networks. At this point, the P(3HB-co-4HB) mixtures of different 4HB contents can be obtained by melt blending, instead of by controlling the 4HB feed in bacterial biosynthesis. From the point of view of the industrial mass production of P(3HB-co-4HB), it may be easier and more useful to control the 4HB content in P(3HB-co-4HB) by melt blending, instead of by bacterial biosynthesis, which involves a complex process. In the copolymer, crystallizable 4HB exists in the main chain of crystalline P(3HB-co-4HB), whereas in the mixtures, a blended noncrystalline P(3HB-co-4HB), having 4HB segments, coexists as a co-domain in the crystalline P(3HB-co-4HB) domain. This difference of micro and macro structure, between the biosynthesized copolymer and their mixture, is expected to have a different effect on the physical properties of P(3HB-co-4HB). Our study focused on the change of physical properties of P(3HB-co-4HB) mixtures by melt blending and compared this with those of bacterial biosynthesis.

### 3.2. Thermal Properties of P(3HB-co-4HB) Mixtures

**Figure 3:**
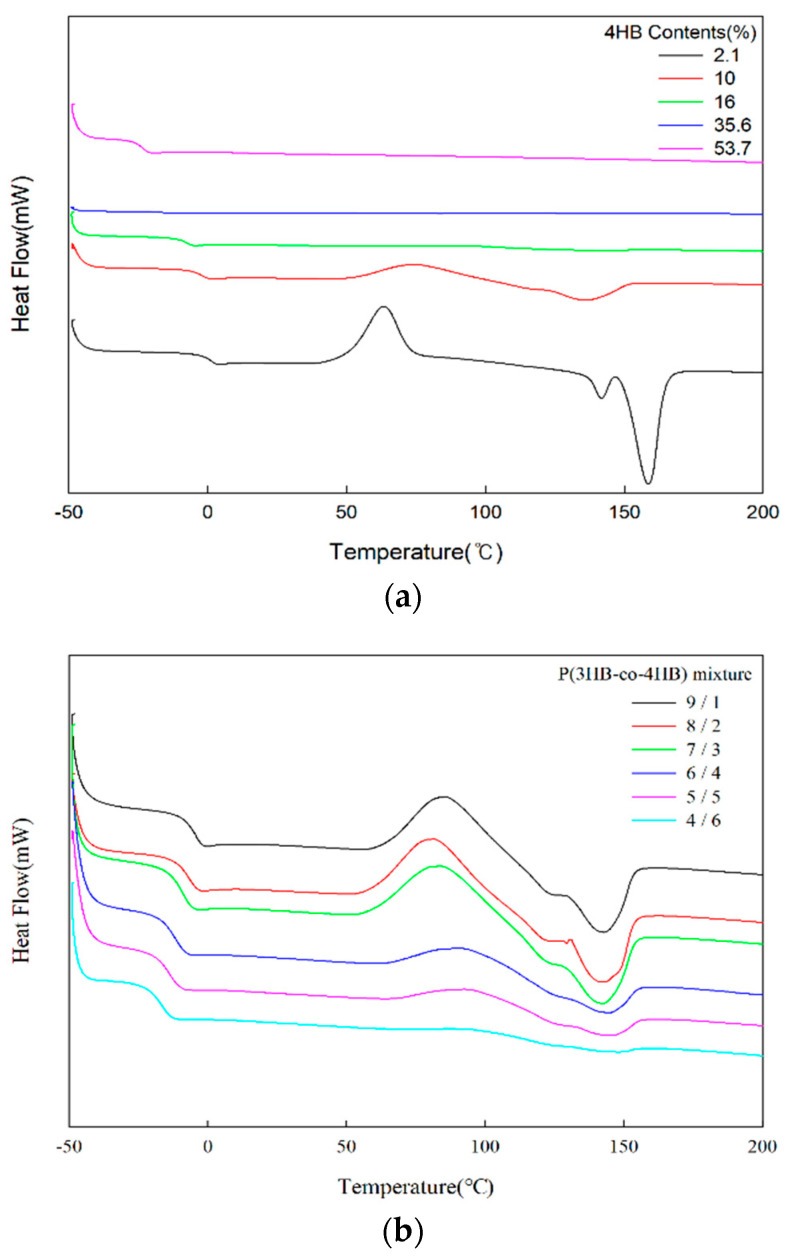
The DSC thermograms of: (**a**) P(3HB-co-4HB) and (**b**) P(3HB-co-4HB) mixtures (cooling rate/heating rate; 20 °C/min).

The secondary heating DSC thermograms of P(3HB-co-4HB) and P(3HB-co-4HB) mixtures are shown in Figure 3. The P(3HB-co-4HB) copolymer is crystalline only up to 16% 4HB, while the P(3HB-co-4HB) mixtures of 4HB contents 14.4–45% all show cold crystallization and melting peaks. P(3HB-co-4HB) is a random copolymer and the chain randomness increases with the increase in the 4HB content to hinder the crystallization, and it becomes noncrystalline P(3HB-co-4HB) at 4HB contents above 16%. However, in the case of blends of crystalline P(3HB-co-4HB) and noncrystalline P(3HB-co-4HB), the increase in 4HB content does not affect the randomness of the copolymer chain and the crystallization of the crystalline P(3HB-co-10% 4HB) is not influenced by the presence of the noncrystalline P(3HB-co-53.7% 4HB) resulting in the appearance of the cold crystallization and melting peaks with change in only the peak areas. This demonstrates that 4HB content, which influences the flexibility and biodegradability of P(3HB-co-4HB) copolymers [35], can be high while retaining the crystallinity and mechanical properties in the case of P(3HB-co-4HB) mixtures from melt blending. Multiple melting peaks are founded in both copolymer and their mixtures. This can be explained by micro-phase separation behavior. The random P(3HB-co-4HB) copolymer may have two phases, a 3HB rich area and a 4HB rich area, each having a different melting temperature. The difference of chain flexibility results in a high melting temperature for the 3HB rich area, and a low melting temperature for the 4HB rich area.

**Figure 4:**
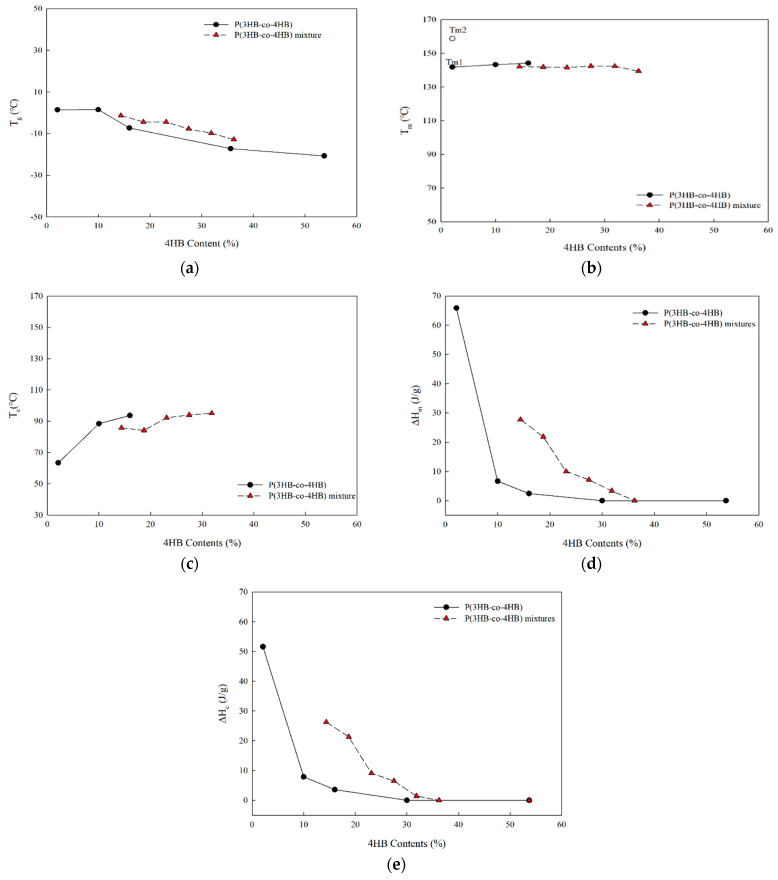
Thermal properties of P(3HB-co-4HB) and P(3HB-co-4HB) mixtures; (**a**) glass transition temperature; (**b**) melting temperature; (**c**) crystallization temperature; (**d**) melting enthalpy; (**e**) crystallization enthalpy.

Figure 4 shows the effect of 4HB content on the glass transition temperature, crystallization temperature and enthalpy, melting temperature and enthalpy of P(3HB-co-4HB) and P(3HB-co-4HB) mixtures. The P(3HB-co-4HB) mixtures prepared from copolymers of different 4HB contents show a single glass transition as a result of their miscibility, and as the 4HB content increases with increase in the composition of flexible P(3HB-co-53.7% 4HB), a decrease in the glass transition temperature occurs. Compared to a copolymer with similar 4HB content, a higher glass transition temperature can be seen in the mixture as the flexibility of the mixture physically blended with the P(3HB-co-53.7% 4HB) chain is lower than that of the copolymer chain with the corresponding amount of 4HB units.

The melting points of mixtures in Figure 4b exhibit negligible change in 4HB content and the melting peak occurs at, or even above, the 4HB content of 30%, while it does not appear in the P(3HB-co-35.6% 4HB) copolymer. This suggests that the increase in the 4HB content in the copolymer increases the randomness of the copolymer, while the increase in the 4HB content in the mixture through physical mixing does not greatly affect the crystallization of the P(3HB-co-10% 4HB) in the mixture. The appearance of the melting peak is apparently due to the crystallization in the cooling stage. Crystallization temperature of the mixtures in Figure 4c are similar to the copolymer of equivalent 4HB content and crystallization enthalpy and melting enthalpy are much higher, with crystallization occurring at 4HB contents even greater than 30% (Figure 4d,e). Therefore, crystalline P(3HB-co-4HB) mixtures of higher 4HB content can be prepared, while crystalline P(3HB-co-4HB) copolymer cannot be obtained at higher 4HB content.

### 3.3. Rheological Properties of P(3HB-co-4HB) Mixtures

**Figure 5:**
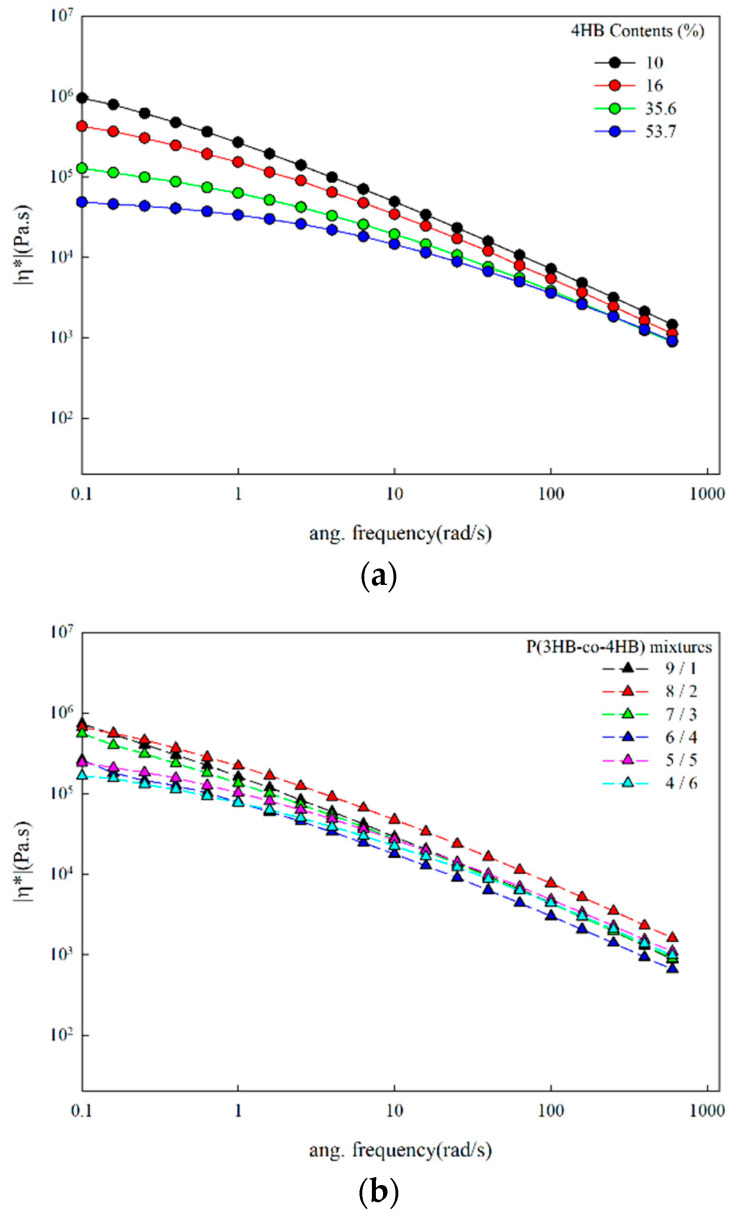
Changes in the complex viscosity with shear rate at 140 °C: (**a**) P(3HB-co-4HB); (**b**) P(3HB-co-4HB) mixtures.

The complex viscosity at 140 °C of P(3HB-co-4HB) and their mixtures are shown in Figure 5. The complex viscosity of P(3HB-co-4HB) mixtures decrease with the shear rate, which is representative of non-Newtonian behavior similar to the P(3HB-co-4HB) copolymers. The complex viscosity is related with chain flexibility and molecular weight. The complex viscosity of P(3HB-co-16% 4HB), having a higher molecular weight and 4HB content compared to P(3HB-co-10% 4HB), is lower than that of P(3HB-co-10% 4HB). This means that the effect of chain flexibility on complex viscosity is dominant and controls the complex viscosity of P(3HB-co-4HB), when compared with the effect of the molecular weight. Increasing the 4HB content in the copolymer resulted in lowering of the complex viscosity in both the copolymer and their mixtures, however, the viscosity change in the 4HB content at a low shear rate is smaller compared to the copolymers in the case of copolymer mixtures of higher P(3HB-co-53.7% 4HB) composition, and thus have higher 4HB contents. In the case of copolymer mixtures, the viscosity decreases due to the physical melt blending with P(3HB-co-53.7% 4HB) having a relatively low viscosity, however, the decrease in 4HB content is smaller compared to in P(3HB-co-4HB) copolymers where the 4HB units are in the main chain.

**Figure 6:**
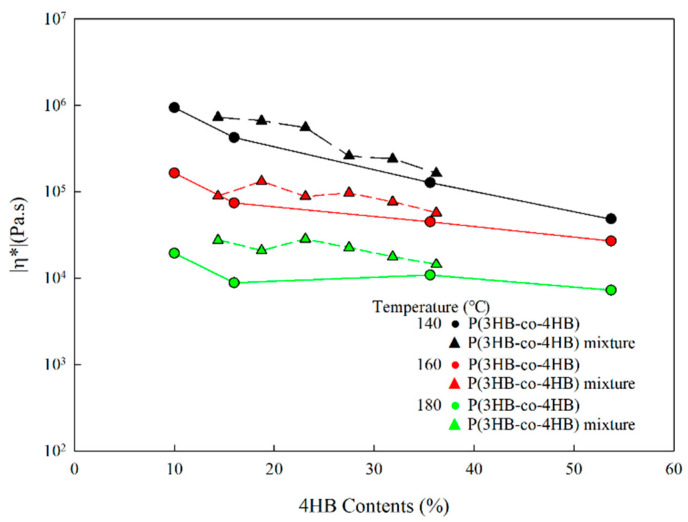
Changes in the complex viscosity of P(3HB-co-4HB) and P(3HB-co-4HB) mixtures with 4HB content at 0.1 rad/s.

**Figure 7:**
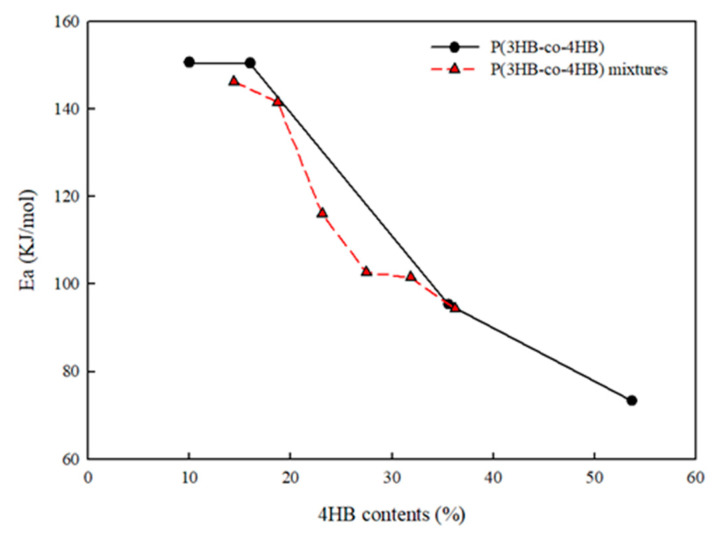
Changes in the activation energy of P(3HB-co-4HB) and P(3HB-co-4HB) mixtures with various 4HB content.

The changes in the viscosity at 0.1 rad/s with 4HB content of P(3HB-co-4HB) copolymers and P(3HB-co-4HB) copolymer mixtures in Figure 6 show that, for same 4HB contents, the P(3HB-co-4HB) mixtures exhibit a higher viscosity than the P(3HB-co-4HB) copolymers. It seems that difference in viscosity between the copolymer and their mixture is more pronounced at a high processing temperature, due to the high chain mobility of 4HB in the copolymer. Changes in the activation energy with 4HB content in P(3HB-co-4HB) copolymers and P(3HB-co-4HB) copolymer mixtures, as shown in Figure 7, show that the activation energy is highest in the case of P(3HB-co-10% 4HB), where the main chain is relatively stiff and decreases as the content of the relatively flexible 4HB in the copolymer increases. Activation energy in the Arrhenius equation reflects the effect of temperature on the mobility of chain. As high activation energy reflects difficult chain motion, the data shows that the increase in 4HB content in P(3HB-co-4HB) increases the chain flexibility. The activation energy of the P(3HB-co-4HB) copolymer mixture also decreases with an increase in the composition of the relatively flexible P(3HB-co-53.7% 4HB). Although the copolymer exhibits a significant decrease in the activation energy at 16% 4HB content, the copolymer mixture exhibits a linear decrease with 4HB content, suggesting that the chain flexibility may be affected by whether 4HB and 3HB units are chemically connected or physically mixed.

**Figure 8:**
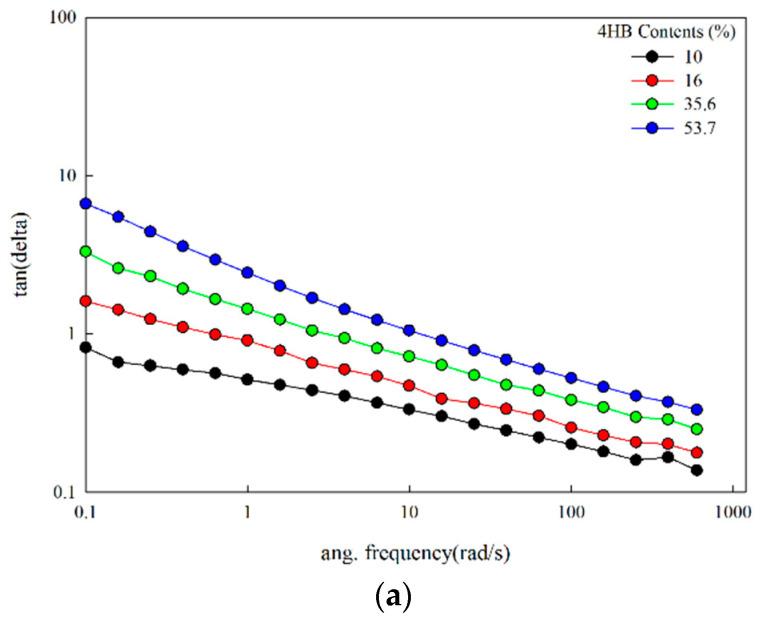

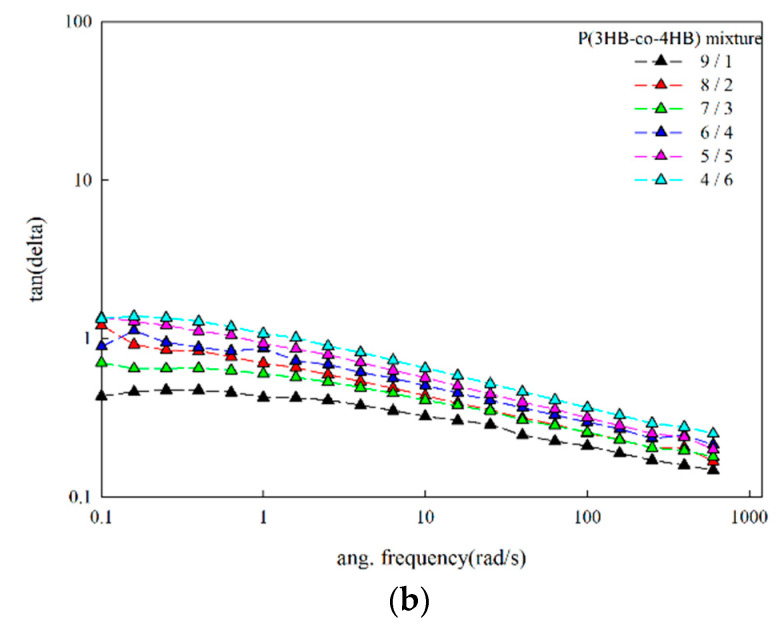
Changes in loss tangent with shear rate at 140 °C: (**a**) P(3HB-co-4HB); (**b**) P(3HB-co-4HB) mixtures.

The loss tangent of P(3HB-co-4HB) copolymers and P(3HB-co-4HB) copolymer mixtures at 140 °C, shown in Figure 8, both increase with an increase in 4HB content. The P(3HB-co-4HB) with high 4HB content shows much less significant frequency dependence of the loss tangent. This indicated that P(3HB-co-4HB) with a high 4HB content has a higher elasticity. It is well known that the melt strength is related to the elasticity of polymers. The loss tangent is known to be inversely proportional to the melt strength [36,37]. The increase in 4HB content increases the chain flexibility and thus decreases the melt strength to decrease the melt processibility, where pressure is applied to form the shape. As can be seen in Figure 8, the loss tangent values of the mixtures are similar to P(3HB-co-10% 4HB), close to 1.0, although there is a slight difference depending on the composition. The lowest loss tangent was obtained with 9/1 composition in the mixtures. The changes in the loss tangent with the increase in the 4HB content is much smaller in the copolymer mixtures compared with the copolymers allowing minimal change in processibility in the case of the copolymer mixtures at the same 4HB content, suggesting that using the copolymer mixtures instead of copolymers of the same 4HB content, is more advantageous for melt processibility.

**Figure 9:**
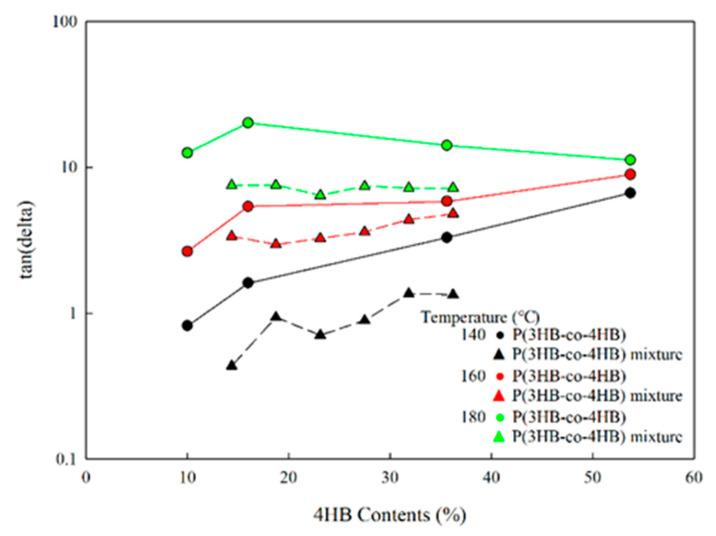
Changes in the loss tangent of P(3HB-co-4HB) and P(3HB-co-4HB) mixtures with 4HB content at 0.1 rad/s.

Figure 9 shows the change in loss tangent with 4HB content at shear rate 0.1 rad/s for P(3HB-co-4HB) and the copolymer mixtures at a different processing temperature. The lower the processing temperature and the lower the 4HB content, the more the loss tangent decreases to result in higher melt strengths and the copolymer mixtures show a lower loss tangent compared to the copolymers, irrespective of the processing temperature or 4HB content. The change in the loss tangent with the increase in 4HB content or processing temperature can be minimized by using copolymer mixtures. This is due to the higher chemically bonded 4HB content of the P(3HB-co-4HB) copolymers, which results in a high increase in tanδ and affect the processibility, while the increase in 4HB content of P(3HB-co-4HB) mixtures, through the physical mixing of P(3HB-co-10% 4HB) and P(3HB-co-53.7% 4HB), does not affect the processibility of P(3HB-co-10% 4HB). From this result, it is found that the enhancement of melt processability could be obtained using P(3HB-co-4HB) mixtures at a low processing temperature.

### 3.4. Mechanical Properties of P(3HB-co-4HB) Mixtures

**Figure 10:**
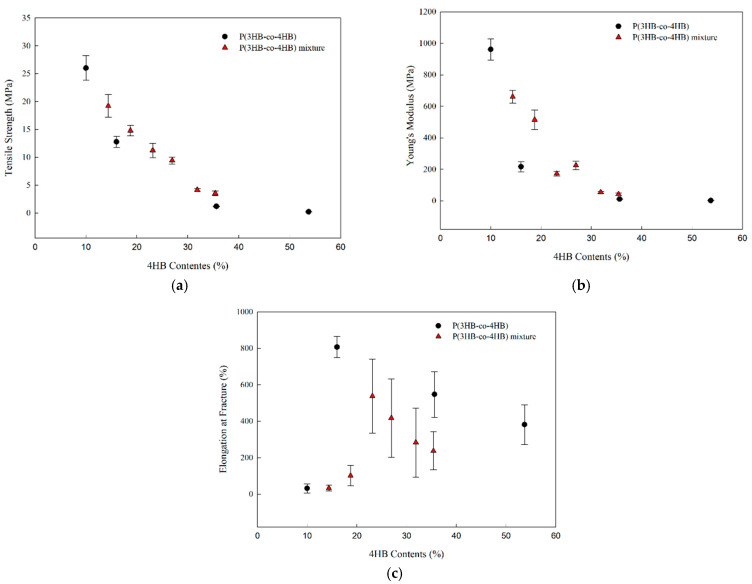
Effect of 4HB content on the mechanical properties of P(3HB-co-4HB) mixtures: (**a**) tensile strength; (**b**) Young’s modulus; (**c**) elongation at break.

Figure 10 shows the effect of 4HB content on the Young’s modulus, tensile strength, and elongation at break of P(3HB-co-4HB) copolymers and P(3HB-co-4HB) copolymer mixtures. The Young’s modulus and tensile strength decrease with the increase in 4HB content and the elongation at break increases to a certain 4HB content, then decreases. It seems that the change in the mechanical properties of P(3HB-co-4HB) and mixtures as a function of 4HB content is due to the flexibility of the 4HB chain in both crystalline P(3HB-co-4HB) and noncrystalline P(3HB-co-4HB). This is observed in both P(3HB-co-4HB) copolymers and P(3HB-co-4HB) copolymer mixtures. Tensile strength and Young’s modulus have an intimate relationship with crystallinity. As the 4HB content increases, crystallinity of both copolymers and copolymer mixtures decrease and spherulites from the crystallization of the 3HB segments become smaller, along with the relatively low crystallization rate of the 4HB segments, which also make the spherulites smaller to decrease the interfacial area of impingement and thus increase the elongation at break. The elongation at break of copolymers decrease in the 4HB contents above 30%, and that of the copolymer mixtures decrease in the 4HB contents above 25%. Above a certain composition, the increase in the P(3HB-co-53.7% 4HB) composition does not increase the elongation at break, but decreases it. The optimum composition for lowering the brittle character of P(3HB-co-10% 4HB) in this study was 7/3, however, for increasing the elongation at break, increasing the 4HB content in P(3HB-co-4HB) copolymer was more effective compared to increasing it in the copolymer mixtures. This suggests that the influence on the elongation at break of 4HB in the copolymer mixtures is lower than that in the copolymers, where they are chemically bound. The processibility of P(3HB-co-4HB) could be enhanced by preparing copolymer mixtures, however, there is a limitation in enhancing the mechanical properties.

## 4. Conclusions

The change in the properties, with variation in the 4HB content of the P(3HB-co-4HB) copolymers and the mixtures of crystalline and noncrystalline P(3HB-co-4HB) copolymers, have been studied. It is found that crystalline P(3HB-co-4HB), with high 4HB content, can be prepared by melt blending. An increase in the 4HB content of P(3HB-co-4HB) copolymers increases the flexibility of the polymer chains to decrease the glass transition temperature and also increases the randomness to decrease the crystallinity. The P(3HB-co-4HB) copolymer mixtures show a higher glass transition temperature and also maintain crystallinity compared to copolymers of the same 4HB content. An increase in the 4HB content of P(3HB-co-4HB) copolymers decreases processibility due to a decrease in the melt viscosity, and an increase in the loss tangent. While in the case of P(3HB-co-4HB), the decrease in the copolymer mixtures’ melt viscosity is relatively smaller, and the increase in the loss tangent is also smaller, such that a P(3HB-co-4HB) copolymer mixture exhibiting a minimal decrease in the processibility with an increase in 4HB content can be prepared. The P(3HB-co-4HB) copolymers and mixtures of crystalline and noncrystalline P(3HB-co-4HB) both exhibited a decrease in tensile strength and Young’s modulus with an increase in 4HB content, however, an adequate increase in the 4HB content increases the flexibility of the polymer chains to increase the elongation at break.

## Figures and Tables

**Table 1:** 4HB Contents and molecular weight (*M*_W_) of P(3HB-co-4HB) used in this study.

Name	4HB (%)	*M*_W_ (k)
P(3HB-co-2.1% 4HB)	2.1	200
P(3HB-co-10% 4HB)	10	600
P(3HB-co-16% 4HB)	16	1000
P(3HB-co-35.6% 4HB)	35.6	580
P(3HB-co-53.7% 4HB)	53.7	695

## Data Availability

Data are in the authors’ possession.

## References

[1] Kanesawa Y., Tanahashi N., Doi Y. (1994). Enzymatic degradation of microbial poly(3-hydroxyalkanoates). Polym. Degrad. Stab..

[2] Chen G.-Q. (2010). Introduction of bacterial plastics PHA, PLA, PBS, PE, PTT, and PPP. Plastics from Bacteria: Natural Functions and Applications.

[3] Norhafini H., Huong K.H., Amirul A.A. (2019). High PHA density fed-batch cultivation strategies for 4HB-rich P(3HB-co-4HB) copolymer production by transformant Cupriavidus malaysiensis USMAA1020. Int. J. Biol. Macromol..

[4] Chanprateep S., Buasri K., Muangwong A.P. (2010). Utiswannakul, Biosynthesis and biocompatibility of biodegradable poly(3-hydroxybutyrate-co-4-hydroxybutyrate). Polym. Degrad. Stab..

[5] Chen G.Q. (2009). A microbial polyhydroxyalkanoates (PHA) based bio- and materials industry. Chem. Soc. Rev..

[6] Ong S.Y., Chee J.Y., Sudesh K. (2017). Degradation of Polyhydroxyalkanoate (PHA): A Review. J. Sib. Fed. Univ. Biol..

[7] Raquez J.M., Habili Y., Murariu M., Dubois P. (2013). Polylactide (PLA)-based nanocomposites. Prog. Polym. Sci..

[8] Lim L.T., Auras R., Rubino M. (2008). Processing technologies for poly(lactic acid). Prog. Polym. Sci..

[9] Liu L.F., Yu J.Y., Cheng L.D., Yang X.J. (2009). Biodegradability of poly(butylene succinate) (PBS) composite reinforced with jute fibre. Polym. Degrad. Stab..

[10] Xu D.J., Guo B.H. (2010). Poly(butylene succinate) and its copolymers: Research, development and industrialization. Biotechnol. J..

[11] El-Hadi A., Schnabel R., Straube E., Müller G., Henning S. (2002). Correlation between degree of crystallinity, morphology, glass temperature, mechanical properties and biodegradation of poly(3-hydroxyalkanoate) PHAs and their blends. Polym. Test..

[12] Erich M., Hannes G., Maximilian L. (2018). PHB-bio based and biodegradable replacement for PP: A Review. Nov. Tech. Nutr. Food Sci..

[13] Savenkova L., Gercberga Z., Nikolaeva V., Dzene A., Bibers I., Kalnin M. (2000). Mechanical properties and biodegradation characteristics of PHB-based films. Process. Biochem..

[14] Patricia P.S.O., Pereira F.V., Santos M.C., Souza P.P., Roa J.P.B., Orefice R.L. (2013). Increasing the elongation at break of polyhydroxybutyrate biopolymer: Effect of cellulose nanowhiskers on mechanical and thermal properties. J. Appl. Polym. Sci..

[15] Panaitescu D.M., Popa M.S., Raditoiu V., Frone A.N., Sacarescu L., Gabor A.R., Teodorescu M. (2021). Effect of calcium stearate as a lubricant and catalyst on the thermal degradation of poly (3-hydroxybutyrate). Int. J. Biol. Macromol..

[16] Pachekoski W.M., Dalmolin C., Agnelli J.A.M. (2013). The Influence of the Industrial Processing on the Degradation of Poly(hidroxybutyrate)—PHB. Macromol. Res..

[17] Koyama N., Doi Y. (1995). Morphology and biodegradability of a binary blend of poly ((R)-3-hydroxybutyric acid) and poly ((R, S)-lactic acid). Can. J. Microbiol..

[18] Wang Y.X., Huang G.C., Luo L.Q. (2016). Thermal Properties of Poly(Lactic Acid) Modified Poly(3-Hydroxybutyrate-co-4-Hydroxbutyrate). Mater. Sci. Forum.

[19] Kennouche S., Moigne N.L., Kaci M., Quantin J.C., Caro-Bretelle A.S., Delaite C., Lopez-Cuesta J.M. (2016). Morphological characterization and thermal properties of compatibilized poly(3-hydroxybutyrate-co-3-hydroxyvalerate) (PHBV)/poly(butylene succinate) (PBS)/halloysite ternary nanocomposites. Eur. Polym. J..

[20] Bian Y., Han L., Han C., Lin H., Zhang H., Bian J., Dong L. (2014). Intriguing crystallization behavior and rheological properties of radical-based crosslinked biodegradable poly (3-hydroxybutyrate-co-4-hydroxybutyrate). CrystEngComm.

[21] Li M., Li Z.Q., Xu J., Wei D.S., Zhu H.W., Li D. (2011). Thermal property, morphology, mechanical and rheological properties of a modified bio-polymers prepared by blending poly (3-hydrobutyrate-co-4-hydrobutyrate) with chain extenders. Open J. Adv. Mater. Res..

[22] Xiang H., Chen Z., Zheng N., Zhang X., Zhu L., Zhou Z., Zhu M. (2019). Melt-spun microbial poly (3-hydroxybutyrate-co-3-hydroxyvalerate) fibers with enhanced toughness: Synergistic effect of heterogeneous nucleation, long-chain branching and drawing process. Int. J. Biol. Macromol..

[23] Fei B., Chen C., Chen S., Peng S., Zhuang Y., An Y., Dong L. (2004). Crosslinking of poly [(3-hydroxybutyrate)-co-(3-hydroxyvalerate)] using dicumyl peroxide as initiator. Polym. Int..

[24] Rupp B., Ebner C., Rossegger E., Slugovc C., Stelzer F., Wiesbrock F. (2010). UV-induced crosslinking of the biopolyester poly (3-hydroxybutyrate)-co-(3-hydroxyvalerate). Curr. Green Chem..

[25] Bhubalan K., Lee W.H., Loo C.Y., Yamamoto T., Tsuge T., Doi Y., Sudesh K. (2008). Controlled biosynthesis and characterization of poly (3-hydroxybutyrate-co-3-hydroxyvalerate-co-3-hydroxyhexanoate) from mixtures of palm kernel oil and 3HV-precursors. Polym. Degrad. Stab..

[26] Loo C.Y., Sudesh K. (2007). Biosynthesis and native granule characteristics of poly (3-hydroxybutyrate-co-3-hydroxyvalerate) in Delftia acidovorans. Int. J. Biol. Macromol..

[27] Liu Q., Luo G., Zhou X.R., Chen G.Q. (2011). Biosynthesis of poly (3-hydroxydecanoate) and 3-hydroxydodecanoate dominating polyhydroxyalkanoates by β-oxidation pathway inhibited Pseudomonas putida. Metab. Eng..

[28] Kim J.S., Lee B.H., Kim B.S. (2005). Production of poly (3-hydroxybutyrate-co-4-hydroxybutyrate) by Ralstonia eutropha. Biochem. Eng. J..

[29] Saito Y., Nakamura S., Hiramitsu M., Doi Y. (1996). Microbial synthesis and properties of poly (3-hydroxybutyrate-co-4-hydroxybutyrate). Polym. Int..

[30] Valentin H.E., Dennis D. (1997). Production of poly (3-hydroxybutyrate-co-4-hydroxybutyrate) in recombinant Escherichia coli grown on glucose. J. Biotechnol..

[31] Al-Kaddo K.B., Mohamad F., Murugan P., Tan J.S., Sudesh K., Samian M.R. (2020). Production of P(3HB-co-4HB) copolymer with high 4HB molar fraction by Burkholderia contaminans Kad1 PHA synthase. Biochem. Eng. J..

[32] Yun O.Y., Min X., Li Y. (2011). Properties analysis of biodegradable material P(3HB-co-4HB). Open J. Adv. Mater. Res..

[33] Wen X., Lu X., Peng Q., Zhu F., Zheng N. (2012). Crystallization behaviors and morphology of biodegradablepoly(3-hydroxybutyrate-co-4-hydroxybutyrate). J. Therm. Anal. Calorim..

[34] Omura T., Gato T., Maehara A., Kimura S., Abe H., Iwata T. (2021). Thermal degradation behavior of poly [(R)-3-hydroxybutyrate-co-4-hydroxybutyrate]. Polym. Degrad. Stab..

[35] Doi Y., Segawa A., Kunioka M. (1990). Biosynthesis and characterization of poly(3-hydroxybutyrate-co-4-hydroxybutyrate) in Alcaligenes eutrophus. Int. J. Biol. Macromol..

[36] Yang S.Z., Madbouly S.A., Schrader J.A., Grewell D., Kessler M.R., Graves W.R. (2015). Processing and characterization of bio-based poly (hydroxyalkanoate)/poly(amide) blends: Improved flexibility and impact resistance of PHA-based plastics. J. Appl. Polym. Sci..

[37] Harrison G.M., Melik D.H. (2006). Application of degradation kinetics to the rheology of poly(hydroxyalkanoates). J. Appl. Polym. Sci..

